# First report of transient urinary retention after bilateral lumbar ESPB in a patient with FBSS: a case report

**DOI:** 10.3389/fmed.2025.1683055

**Published:** 2025-10-09

**Authors:** Jianzhong Li, Afeng Zhang, Tiankun Shu, Jun Qiao, Lei Duan

**Affiliations:** ^1^Department of Anesthesiology, Norinco General Hospital, Xi'an City, China; ^2^Department of Anesthesiology, Xidian Group Hospital, Xi’an City, China; ^3^Department of Pain Management, Norinco General Hospital, Xi'an City, China; ^4^Department of Anesthesiology, Xi'an Aerospace Hospital Affiliated of Northwest University, Xi'an City, China

**Keywords:** erector spinae plane block, urinary retention, spondylolisthesis, failed back surgery syndrome, case report

## Abstract

The erector spinae plane block (ESPB) is a regional anesthesia technique increasingly used in recent years for postoperative analgesia in thoracic, abdominal, spinal, and hip surgeries. The adoption of this method has been encouraged by its technical simplicity and a low rate of complications. To date, no case reports have described transient urinary retention following lumbar ESPB. Here, we present the case of a 64-year-old male admitted after a fall that resulted in a right hip fracture. He had previously undergone L5/S1 posterior lumbar interbody fusion, which was unsuccessful, resulting in failed back surgery syndrome (FBSS). To alleviate preoperative lumbosacral pain, bilateral ultrasound-guided ESPB was performed at the L5 transverse process level at the bedside, with 20 mL of 0.2% ropivacaine administered on each side. The procedure was uneventful. Approximately 1 h after the block, the patient experienced a strong urge to void but was unable to urinate. Bedside bladder ultrasonography revealed marked bladder distension, and catheterization yielded 700 mL of urine. By the following morning, with the return of lumbosacral pain sensation, the patient regained spontaneous voiding without other neurological deficits. No recurrence occurred until discharge. This case suggests that in patients with a history of spinal surgery and altered paraspinal anatomy, ESPB may result in unintended blockade due to aberrant spread of local anesthetic into the epidural space. Consequently, a comprehensive preprocedural assessment of spinal anatomy and improved postoperative monitoring of lumbosacral plexus function are advised to ensure early detection and management of this rare complication.

## 1 Introduction

The erector spinae plane block (ESPB) is a relatively recent fascial plane block technique, first introduced by Forero et al. in 2016 for the management of thoracic neuropathic pain (1). ESPB involves the injection of local anesthetic into the fascial plane between the erector spinae muscle and the transverse process. Theoretically, it can block adjacent dorsal rami and, to some extent, the ventral rami of spinal nerves (2), to achieve multi-dermatomal analgesia. ESPB is increasingly adopted for perioperative analgesia in thoracic, abdominal, spinal, and even hip surgeries due to its simplicity, safety, and low complication rate (3–5). However, the specific mechanism of action of ESPB remains incompletely understood. Recently, reports of peripheral nerve dysfunction (such as Harlequin syndrome and Priapism) caused by abnormal spread of local anesthetics after ESPB have increasingly drawn clinical attention (6, 7).

To our knowledge, no cases have been reported in which the ESPB inadvertently involves the sacral plexus and leads to urinary retention. We present a detailed report of a patient with a history of failed back surgery syndrome (FBSS) who developed transient urinary retention following bilateral ESPB at the L5 level for lumbosacral analgesia. The case also explores possible underlying mechanisms in light of the patient’s altered spinal anatomy.

## 2 Case report

**Figure 1:**
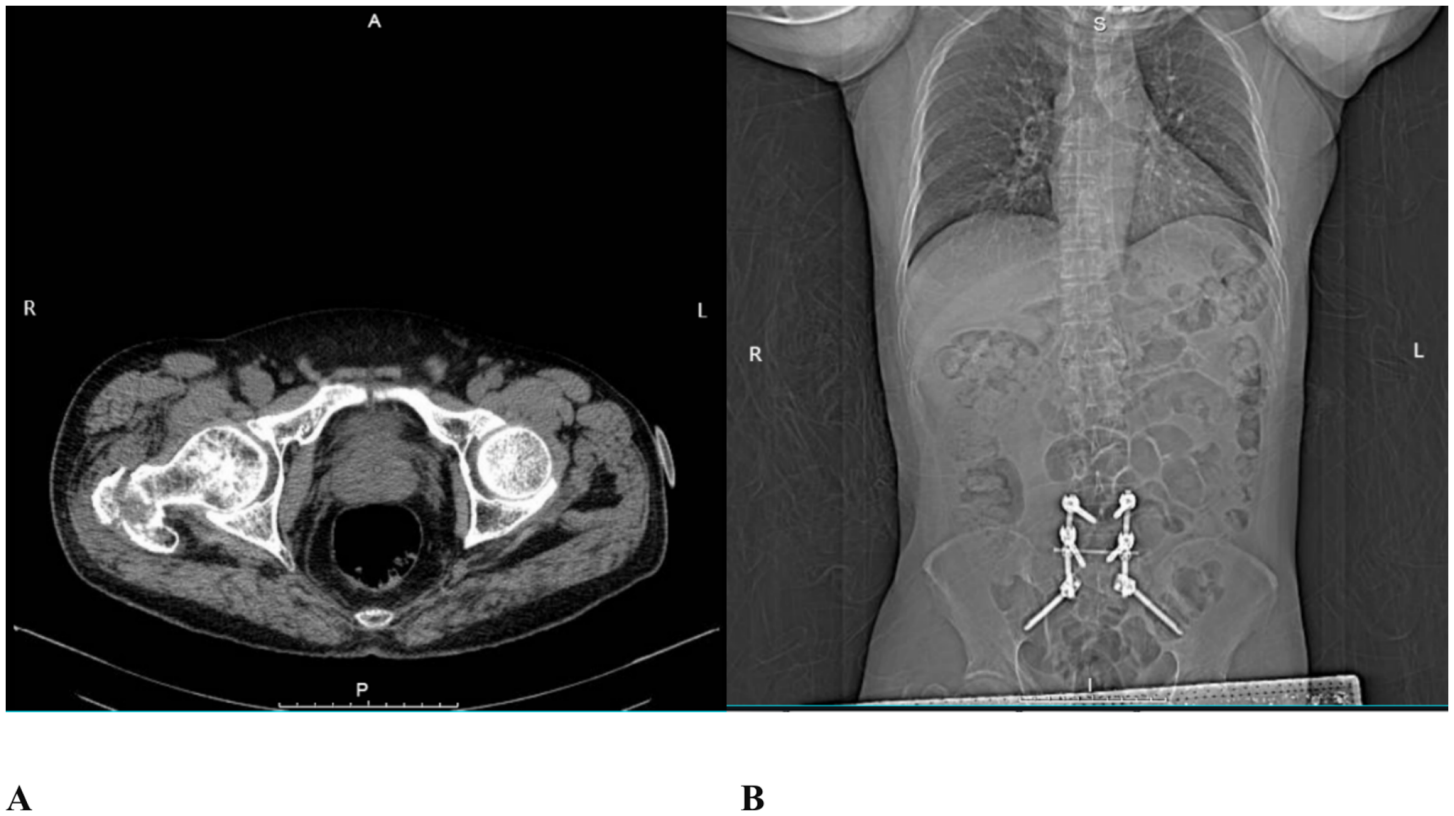
CT images. **(A)** Basal fracture of the right femoral neck; **(B)** Postoperative imaging following prior posterior L5/S1 lumbar interbody fusion.

A 64-year-old male patient (165 cm, 75 kg) was admitted to the hospital with right hip pain and restricted mobility following a fall. Computed tomography (CT) revealed a basal fracture of the right femoral neck (Figure 1A).

In 2021, the patient had undergone posterior lumbar interbody fusion for L5/S1 spondylolisthesis (Figure 1B). Postoperatively, he developed failed back surgery syndrome (FBSS) due to unsuccessful fusion, presenting with persistent pain in the L4 to S1 region. His visual analogue scale (VAS) pain scores ranged from 8 to 10 (on a scale where 0 indicates no pain and 10 represents the worst imaginable pain). Persistent lumbosacral pain resulted in a functionally constrained posture, which led to limited hip joint mobility and difficulty with ambulation. The patient reported that this abnormal posture was a significant contributing factor to his recent fall. Due to FBSS-related chronic pain, he required long-term oral administration of tramadol hydrochloride (100 mg per dose, 3–4 times daily) and the application of transdermal fentanyl patches to maintain his VAS pain score at 5. His medical history includes hypertension for 8 years, type 2 diabetes mellitus for 12 years, and coronary artery disease for 7 years. These conditions have been managed with regular oral medications, including nifedipine, metformin, atorvastatin, and aspirin. The patient had no relevant family history of hereditary disease. He had no previous history of urinary retention and no known history of prostatic disease. Prior to the fall, he lived independently.

**Figure 2:**
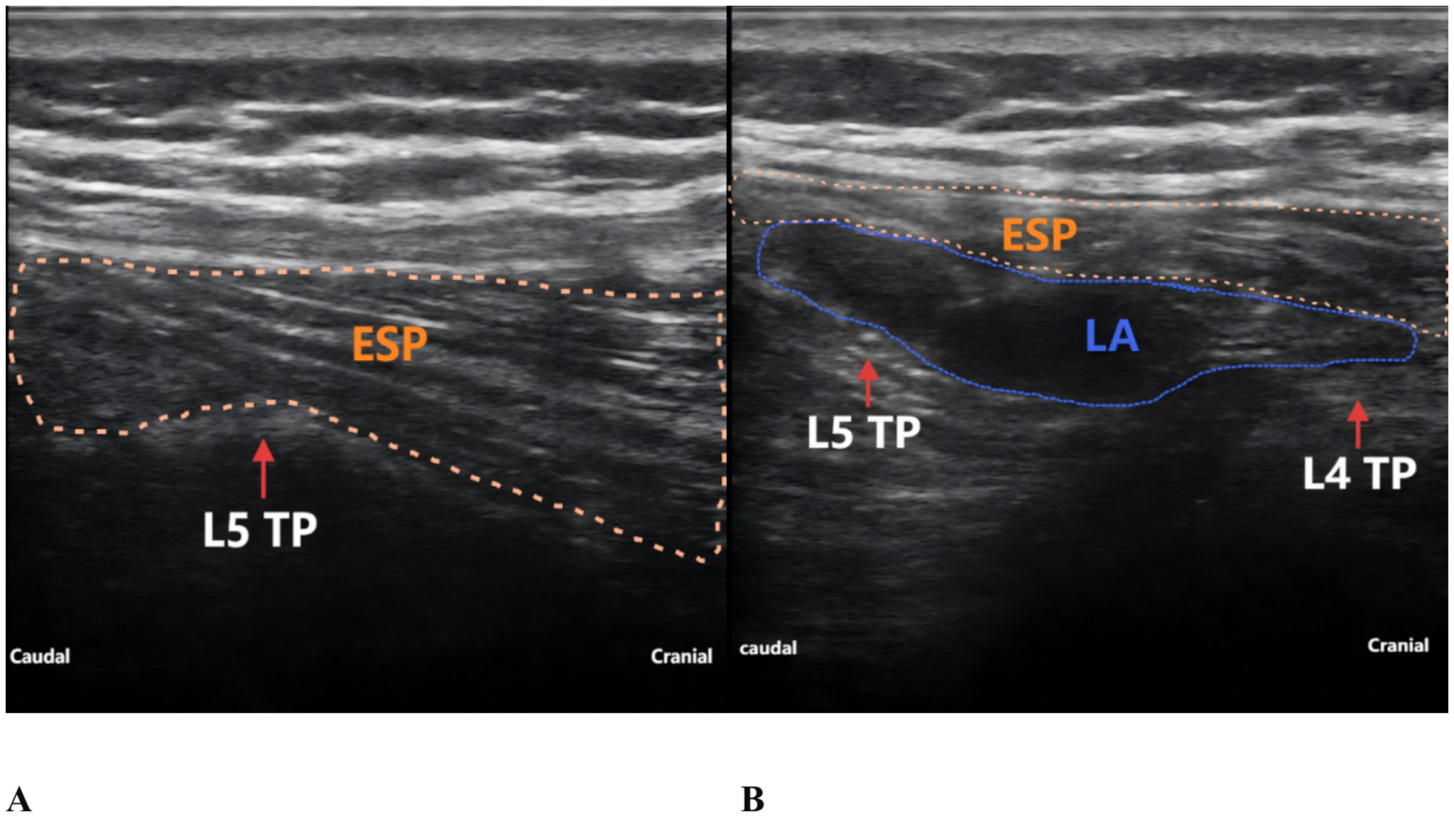
Ultrasound-guided ESPB at the L5 level. **(A)** Before local anesthetic injection. **(B)** After local anesthetic injection. TP, transverse process; ESP, erector spinae muscle; LA, local anesthetic.

**Figure 3:**
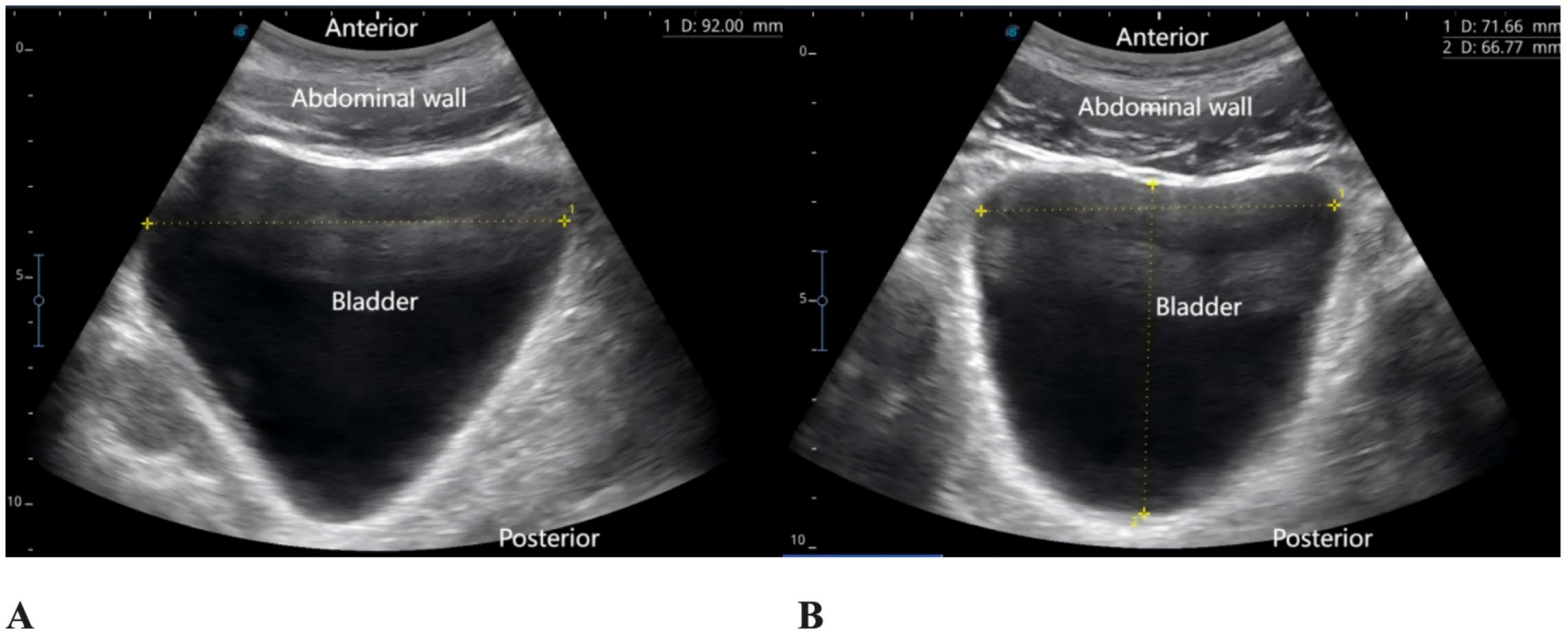
Bladder ultrasound images. **(A)** Sagittal view. **(B)** Transverse view.

The patient reported severe pain in the right hip (VAS score of 9) upon arrival at the emergency department of the hospital (15:00 on May 27, 2025). Physical examination revealed external rotation deformity of the right lower limb, positive tenderness at the midpoint of the inguinal ligament and the greater trochanter, and restricted flexion and extension of the hip joint. Movement of the toes remained intact, and distal perfusion and sensory function of the foot were normal. Urgent laboratory tests—including complete blood count, coagulation profile, and electrocardiogram—showed no significant abnormalities. Subsequently, an ultrasound-guided pericapsular nerve group (PENG) block was administered by the anesthesiologist using 20 mL of 0.2% ropivacaine for right hip analgesia (8). Ten minutes after the block, the patient reported that his hip pain had decreased to a VAS score of 1. At that time, his urinary function was normal, with no signs of urinary retention. He was then transferred to the orthopedic ward for further management. At 18:00 that evening, the patient reported significant relief of right hip pain but complained of severe pain localized to the L5 region, with a VAS score of 9, rendering him unable to lie in the supine position. The anesthesiologist instructed the patient to assume the prone position, and bedside ultrasound scanning of the lumbosacral region was performed. The ultrasound image revealed the presence of spinal fusion hardware from L4 to S1, appearing as a hyperechoic linear structure. A 12 cm longitudinal surgical scar was noted along the midline of the lumbosacral region. The location of the L5 transverse process was identified and marked based on preoperative CT imaging in conjunction with real-time ultrasound guidance. After disinfection of the puncture site, a high-frequency linear transducer (6–13 MHz, SonoSite, USA, Model, S-series) covered with a sterile adhesive drape was placed vertically in the sagittal position on the marked L5 transverse process. 2 mL of 2% lidocaine was used for local anesthesia at the skin puncture site. An 18G × 100 mm needle (Contiplex type D, Braun Melsungen, Germany) was inserted using an in-plane, cranial-to-caudal approach, with the tip advanced into the fascial plane between the erector spinae muscle and the L5 transverse process. After the needle tip contacted the L5 transverse process, 3 mL of normal saline was injected to confirm correct placement. Following negative aspiration for blood or air, 20 mL of 0.2% ropivacaine was injected into the fascial plane between the erector spinae muscle and the transverse process. Ultrasound demonstrated a hypoechoic, elongated spread of the local anesthetic between the deep surface of the erector spinae muscle and the transverse process (Figure 2). The same procedure was performed on the contralateral side. Since this was a routine ultrasound-guided fascial plane block, no injection pressure monitoring device was used. However, during the procedure, the anesthesia assistant observed unusually high resistance to injection. Twenty minutes after the bilateral ESPB, the patient experienced significant alleviation of lumbosacral pain (VAS score of 2), with intact sensory and motor function in both lower limbs. One hour later (at 19:00), the patient complained of a strong urge to void and suprapubic fullness but was unable to urinate. Neurological examination revealed no sensory or motor deficits in either lower limb. A bedside ultrasound by a urologist confirmed significant bladder distension (Figure 3). Urinary catheterization was performed, yielding 700 mL of urine within 5 min, followed by immediate relief of bladder discomfort. At 5:00 the following morning, the patient reported gradual return of sensation in the lumbosacral region and described bladder stimulation caused by the indwelling urinary catheter. After an additional hour of close observation, during which urine output remained normal, the catheter was removed. Spontaneous voiding function was subsequently restored. After the analgesic effects of the PENG and ESPB subsided, the patient continued to receive multimodal analgesia. His baseline regimen included oral tramadol hydrochloride and transdermal fentanyl patches, with additional oral celecoxib administered as required. On hospital day 3, the patient underwent closed reduction and internal fixation for the right femoral neck basal fracture under general anesthesia using a laryngeal mask airway. The patient received our institution’s standard multimodal analgesia regimen for hip surgery, which included oral celecoxib (200 mg once daily) and a patient-controlled intravenous analgesia (PCIA) pump containing oxycodone (100 mg diluted in 0.9% saline to a final concentration of 1 mg/mL, no continuous background infusion, bolus dose 0.03 mg/kg, lockout interval 5 min). No additional regional block was performed, and urinary retention did not recur. Postoperative X-rays on postoperative day 1 and day 5 confirmed satisfactory fracture reduction, and no further episodes of acute urinary retention occurred during this period. The patient was discharged on postoperative day 6. He reported that the regional blocks provided substantial pain relief, allowing him to tolerate positioning and daily care before surgery. Although he experienced some discomfort due to urinary retention, he expressed overall satisfaction with pain management and the recovery process, considering it acceptable compared with the severe pain he had before the blocks. A detailed timeline of the patient’s hospitalization, interventions, and clinical course is summarized in Table 1.

**Table 1:** Timeline of events, interventions, and findings.

Date/Time	Event	Intervention/Findings
May 27, 15:00	Admission after fall, right hip pain (VAS 9)	CT: basal fracture of right femoral neck
May 27, 15:30	PENG block (20 mL 0.2% ropivacaine)	Hip pain relief (VAS 1); urinary function normal
May 27, 18:00	Severe lumbosacral pain (VAS 9)	Bilateral ESPB at L5 level (20 mL 0.2% ropivacaine each side); unusually high injection resistance noted
May 27, 19:00	Acute urinary retention	Strong urge to void, suprapubic fullness; bladder ultrasound: marked distension; catheterization drained 700 mL
May 28, 05:00	Recovery of bladder sensation	Patient perceived catheter-related bladder stimulation; urine output normal during 1-h observation
May 28, 06:00	Catheter removed	Spontaneous voiding function restored; no neurological deficits
May 28, After ESPB/PENG subsided	Continuation of multimodal analgesia	Baseline regimen: oral tramadol hydrochloride and transdermal fentanyl patches; additional oral celecoxib as required
May 29 (Hospital day 3)	Surgery: closed reduction and internal fixation	General anesthesia with LMA; standard multimodal analgesia with PCIA oxycodone (100 mg until discharge)
Post-op day 1–5	Recovery	No recurrence of urinary retention; X-rays confirmed satisfactory fracture reduction
Post-op day 6	Discharge	Pain well controlled; patient satisfied with analgesia and overall recovery

ESPB, erector spinae plane block; PENG, pericapsular nerve group; PCIA, patient-controlled intravenous analgesia; LMA, laryngeal mask airway; VAS, visual analogue scale.

## 3 Discussion

Single-injection ESPB has gained widespread use in perioperative analgesia for various spinal surgeries in recent years, owing to its technical simplicity, clearly identifiable anatomical landmarks, and low complication rate (9). In a meta-analysis of 480 patients, Seok et al. (10) found no significant ESPB-related complications, such as anesthetic toxicity, infection, neurovascular injury, or damage to neuraxial or pulmonary structures. With increasing research into the mechanisms underlying ESPB, evidence suggests that the spread of local anesthetics may extend beyond the fascial plane between the deep surface of the erector spinae muscle and the transverse process (11). This anatomical complexity may result in unintended neural blockade, particularly in specific populations. Amoroso et al. (12) highlighted that at the lumbar level, the unpredictable distribution of local anesthetics following ESPB can lead to inadvertent neuraxial spread. Given the potential alterations in spinal anatomy after lumbosacral fusion surgery and the variability in analgesic efficacy, the exact mechanism of action remains to be elucidated.

We propose that the acute urinary retention observed after the ESPB in this case resembled a low-level bilateral spread typical of epidural anesthesia, which is characterized by selective involvement of the sacral nerves without causing motor blockade. This supports the hypothesis that, in the context of postoperative changes in the lumbosacral region, local anesthetics may have entered the spinal canal but with limited distribution. Two primary mechanisms may explain this phenomenon. First, Chin et al. (13) noted that although local anesthetics primarily spread cranio-caudally within the fascial plane deep to the erector spinae muscle following ESPB, both cadaveric and imaging studies have demonstrated that, under certain conditions, the anesthetic can track through the connective tissue between transverse processes into the paravertebral space, or even pass through the intervertebral foramina to reach the epidural space, resulting in clinical effects akin to epidural blockade. This atypical spread is particularly likely in patients with disrupted anatomical structures. In this specific case, the patient’s surgical history may have led to fascial scarring, disorganized tissue layers, or soft tissue defects in the paraspinal area, all of which could compromise the natural fascial barriers. Consequently, a local anesthetic that would typically remain confined to the transverse process-deep erector spinae interface may have aberrantly spread into the intervertebral foramina or epidural space, exerting effects on sacral nerves (S2–S4). This may have temporarily suppressed detrusor function and reduced control of the external urethral sphincter, leading to temporary urinary retention. Notably, the onset and resolution of urinary retention in this case coincided closely with the previously reported analgesic duration of ESPB (approximately 10 h) (14), further supporting the hypothesis of transient functional blockade of neural structures due to unintended local anesthetic spread.

On the other hand, Amoroso et al. (12) reported two cases of bilateral lower limb sensorimotor impairment following continuous ESPB for postoperative analgesia after spinal fusion surgery. Magnetic resonance imaging (MRI) findings revealed compression of the dural sac, indicating that under conditions of altered spinal anatomy combined with high injection pressure, large volumes, and continuous infusion of local anesthetic, the drug was more likely to enter the epidural space via non-anatomical pathways. In this case, high injection pressure was noted, potentially promoting the spread of local anesthetic along a “low-pressure pathway” instead of within the ideal interfascial plane. This aberrant spread could allow the anesthetic to cross anteriorly over the transverse process and enter the lower spinal canal through the intervertebral foramen, producing effects similar to a sacral plexus block.

Previous case reports have also described autonomic nervous system complications following ESPB. Sullivan et al. reported a case of Harlequin syndrome after ESPB, which was attributed to unintended spread of local anesthetic affecting the thoracic sympathetic chain (6). Similarly, Elkoundi et al. described priapism following ESPB, considered to result from parasympathetic hyperactivity due to sacral autonomic involvement (7). In contrast, our patient developed transient urinary retention, most plausibly explained by blockade of the sacral parasympathetic outflow (S2–S4), which mediates detrusor contraction and initiation of the micturition reflex. Taken together, these observations suggest that ESPB, although generally regarded as a safe fascial plane block, may occasionally result in unanticipated neuraxial or autonomic spread of local anesthetic, with diverse clinical manifestations depending on the level and type of autonomic involvement.

This report has several limitations. First, as the complication occurred following a routine ESPB procedure, the local anesthetic used did not contain a contrast agent, making it impossible to visualize the exact spread of the drug on MRI. Therefore, we could not directly determine whether the local anesthetic entered the spinal canal and resulted in sacral plexus blockade. If available, post-block MRI or CT imaging may help demonstrate whether the dural sac at the affected level was influenced by the local anesthetic, thereby clarifying the diagnosis. Anatomically, normal human micturition relies on parasympathetic innervation from the S2–S4 nerve roots, which control detrusor muscle contraction and initiate the micturition reflex. Additionally, the pudendal nerve, also originating from S2–S4, controls the external urethral sphincter. Any temporary blockade of these segments can lead to urinary retention. Secondly, because ESPB is a fascial plane block, it is theoretically not associated with the risk of direct peripheral nerve injury. Injection pressure monitoring was not utilized in this case. However, in the presence of spinal anatomical abnormalities, even techniques generally regarded as safe—such as ESPB—may result in unintended spread of local anesthetic into the spinal canal under certain conditions (e.g., structural deformities, high injection pressure, or large-volume administration), potentially causing unanticipated neuraxial blockade. Third, intravenous opioid use has been identified as an independent risk factor for urinary retention, primarily through suppression of the micturition reflex (15). However, in this case the patient received only PENG block and ESPB for analgesia prior to the onset of acute urinary retention, without any intravenous opioid administration. Postoperatively, the cumulative oxycodone consumption via the PCIA system was 100 mg until discharge, during which no further episodes of urinary retention occurred. Combined with the absence of previous urinary retention episodes or known prostatic disease, these observations further support our hypothesis that ESPB may have contributed to the transient urinary retention observed in this case.

In conclusion, although cases of ESPB resulting in partial or extensive epidural anesthesia are rare, their incidence may be higher in patients with a history of spinal surgery. Therefore, caution should be exercised when performing ESPB in such patients. We recommend the following precautions: (1) thoroughly assess the anatomical structures and surgical history of the injection site prior to the block; (2) carefully control the volume and concentration of the local anesthetic, routinely use an injection pressure monitoring device, and maintain the injection pressure below 15 PSI (16); and (3) closely monitor lumbosacral nerve function and urinary function after the block. If urinary retention or dysfunction occurs, it is essential to promptly identify and evaluate the possible mechanisms of local anesthetic spread.

## Data Availability

The original contributions presented in the study are included in the article/Supplementary material, further inquiries can be directed to the corresponding author.
