# Supplementary figures and images for: First report of transient urinary retention after bilateral lumbar ESPB in a patient with FBSS: a case report
Source: Front Med (Lausanne). 2025 Oct 9;12:1683055. doi: 10.3389/fmed.2025.1683055 (PMC12546056; doi:10.3389/fmed.2025.1683055)

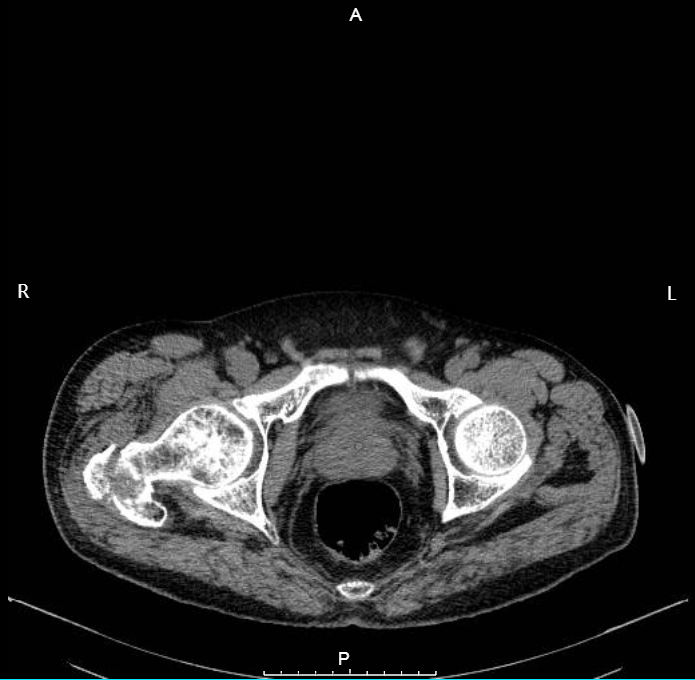

Supplement: Supplementary file 1 [file Data_Sheet_1.zip › Figures/Fig 1A.tif]

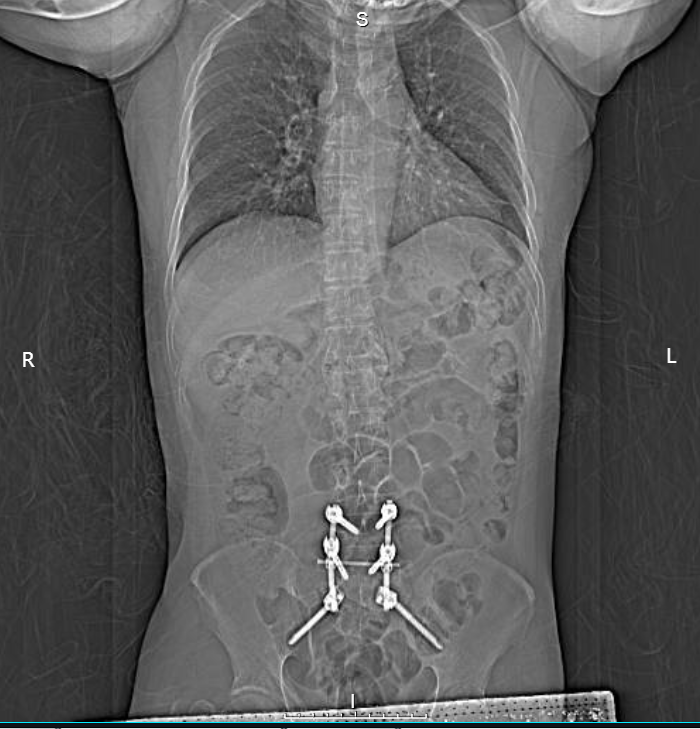

Supplement: Supplementary file 1 [file Data_Sheet_1.zip › Figures/Fig 1B.tif]

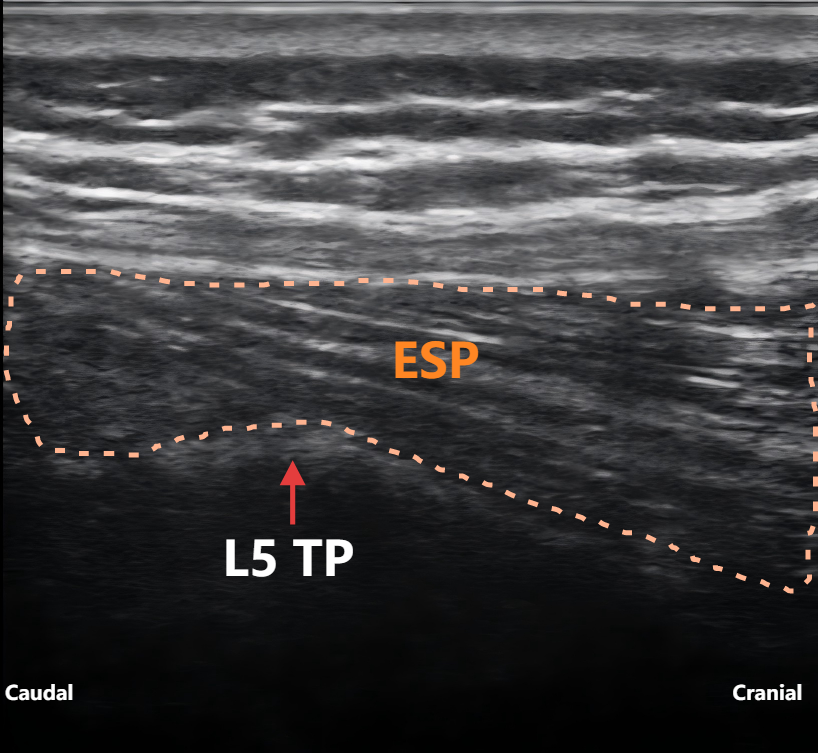

Supplement: Supplementary file 1 [file Data_Sheet_1.zip › Figures/Fig 2A.tif]

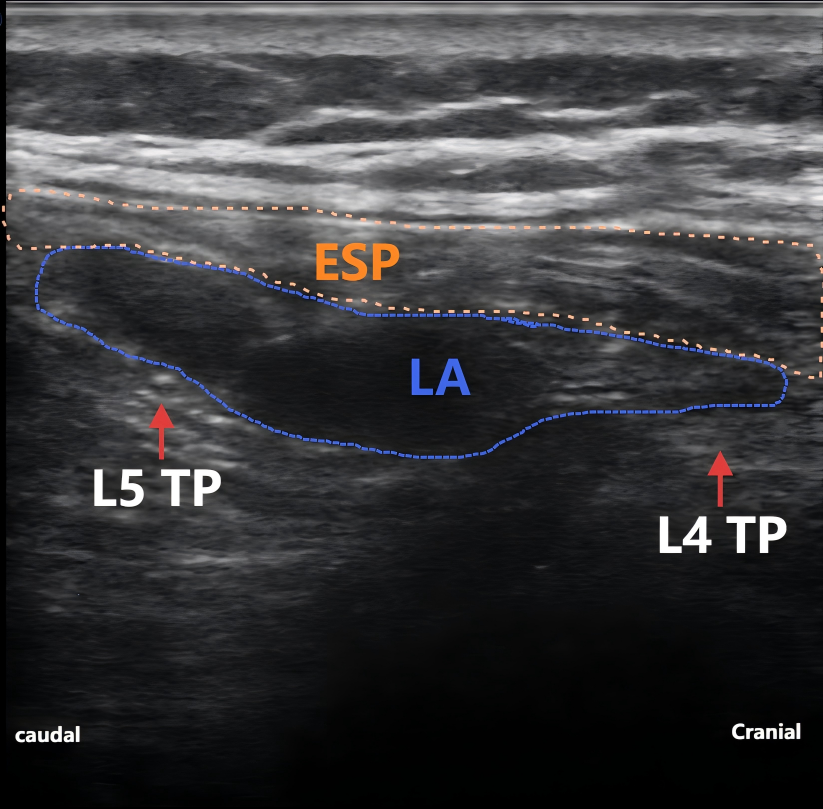

Supplement: Supplementary file 1 [file Data_Sheet_1.zip › Figures/Fig 2B.tif]

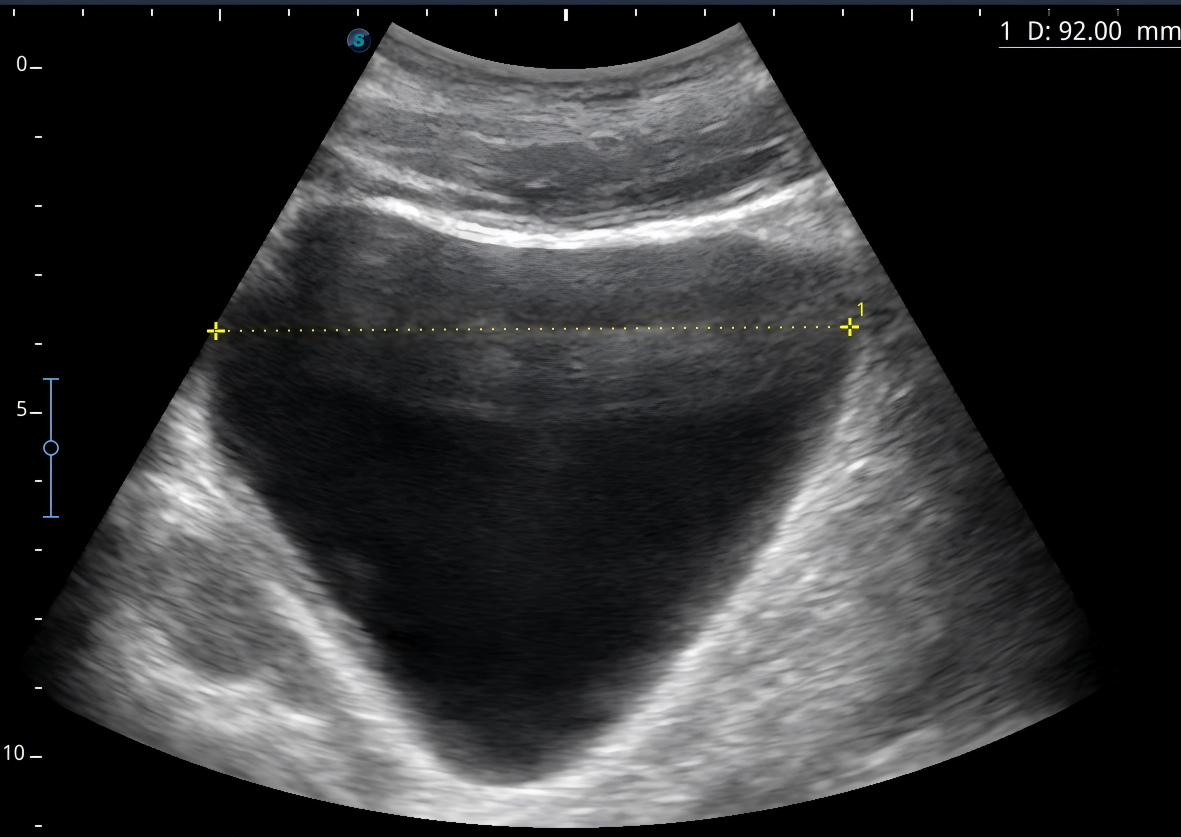

Supplement: Supplementary file 1 [file Data_Sheet_1.zip › Figures/Fig 3A.tif]

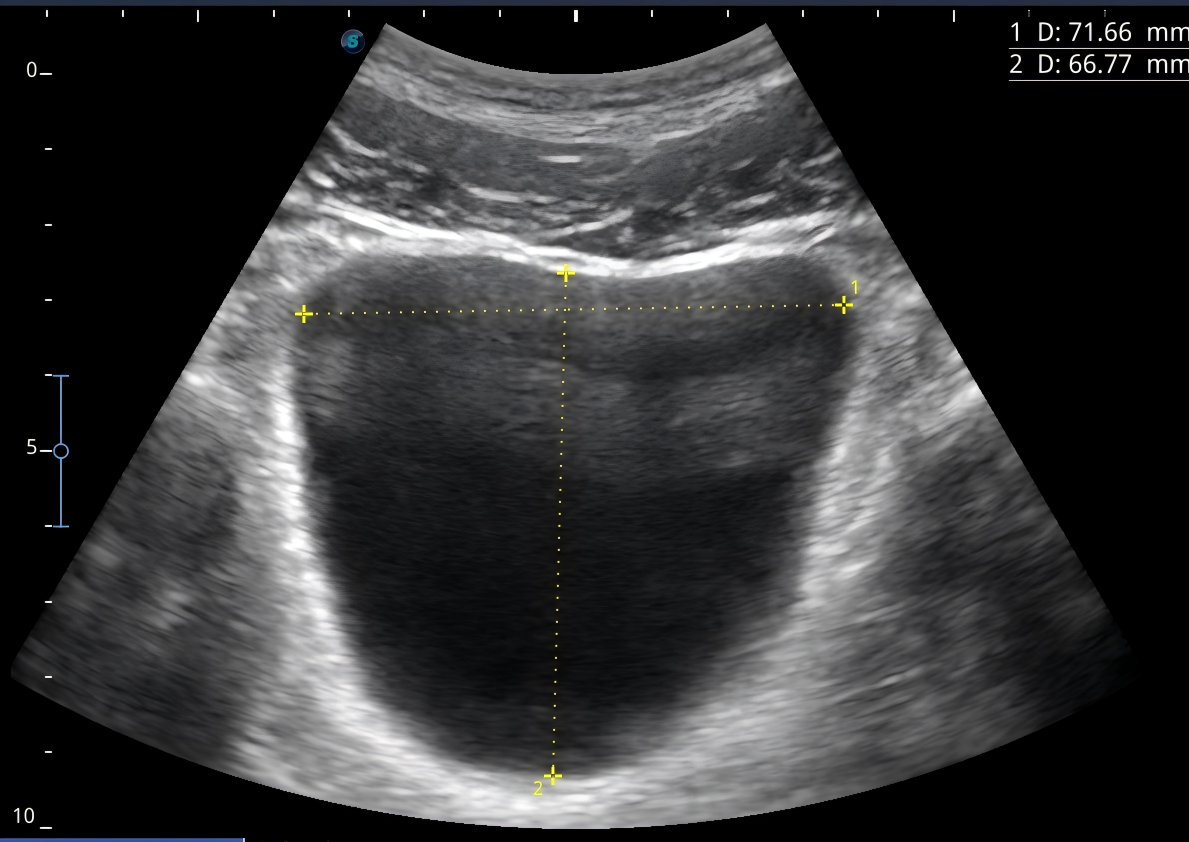

Supplement: Supplementary file 1 [file Data_Sheet_1.zip › Figures/Fig 3B.tif]
