# Supplementary material for: First report of transient urinary retention after bilateral lumbar ESPB in a patient with FBSS: a case report
Source: Front Med (Lausanne). 2025 Oct 9;12:1683055. doi: 10.3389/fmed.2025.1683055 (PMC12546056; doi:10.3389/fmed.2025.1683055)
Supplement: Supplementary file 2 [file Data_Sheet_2.pdf]

CARE
case report guidelines

CARE Checklist of information to include when writing a case report

(cc) BY-NC-ND

Topic	Item	Checklist item description	Reported on Line
Title	1	The diagnosis or intervention of primary focus followed by the words "case report"	Page 1
Key Words	2	2 to 5 key words that identify diagnoses or interventions in this case report, including "case report"	Page 1
Abstract (no references)	3a	Introduction: What is unique about this case and what does it add to the scientific literature?	Page 1-2
	3b	Main symptoms and/or important clinical findings	Page 1-2
	3c	The main diagnoses, therapeutic interventions, and outcomes	Page 1-2
	3d	Conclusion—What is the main "take-away" lesson(s) from this case?	Page 2
Introduction	4	One or two paragraphs summarizing why this case is unique (**may include references**)	Page 2
Patient Information	5a	De-identified patient specific information.	Page 3
	5b	Primary concerns and symptoms of the patient.	Page 3
	5c	Medical, family, and psycho-social history including relevant genetic information	Page 3
	5d	Relevant past interventions with outcomes	Page 3
Clinical Findings	6	Describe significant physical examination (PE) and important clinical findings.	Page 3-4
Timeline	7	Historical and current information from this episode of care organized as a timeline	Page 3-5
Diagnostic Assessment	8a	Diagnostic testing (such as PE, laboratory testing, imaging, surveys).	Page 3
	8b	Diagnostic challenges (such as access to testing, financial, or cultural)	None
	8c	Diagnosis (including other diagnoses considered)	Page 3
	8d	Prognosis (such as staging in oncology) where applicable	Page 5
Therapeutic Intervention	9a	Types of therapeutic intervention (such as pharmacologic, surgical, preventive, self-care)	Page 4-5
	9b	Administration of therapeutic intervention (such as dosage, strength, duration)	Page 4-5
	9c	Changes in therapeutic intervention (with rationale)	Page 4-5
Follow-up and Outcomes	10a	Clinician and patient-assessed outcomes (if available)	Page 5
	10b	Important follow-up diagnostic and other test results	Page 5
	10c	Intervention adherence and tolerability (How was this assessed?)	Page 4-5
	10d	Adverse and unanticipated events	Page 5
Discussion	11a	A scientific discussion of the strengths AND limitations associated with this case report	Page 5-7
	11b	Discussion of the relevant medical literature **with references**.	Page 5-7
	11c	The scientific rationale for any conclusions (including assessment of possible causes)	Page 5-8
	11d	The primary "take-away" lessons of this case report (without references) in a one paragraph conclusion	Page 8
Patient Perspective	12	The patient should share their perspective in one to two paragraphs on the treatment(s) they received	Page 5
Informed Consent	13	Did the patient give informed consent? Please provide if requested	Yes ☑ No ☐
